# NF2/Merlin Inactivation and Potential Therapeutic Targets in Mesothelioma

**DOI:** 10.3390/ijms19040988

**Published:** 2018-03-26

**Authors:** Tatsuhiro Sato, Yoshitaka Sekido

**Affiliations:** 1 Division of Molecular Oncology, Aichi Cancer Center Research Institute, 1-1 Kanokoden, Chikusa-ku, Nagoya 464-8681, Japan; satot@aichi-cc.jp; 2 Department of Cancer Genetics, Nagoya University Graduate School of Medicine, 65, Tsurumai-cho, Showa-ku, Nagoya 464-8603, Japan

**Keywords:** malignant mesothelioma, neurofibromatosis type 2 (*NF2*), merlin, Hippo signaling pathway, PI3K/AKT/mTOR signaling pathway

## Abstract

The neurofibromatosis type 2 (*NF2*) gene encodes merlin, a tumor suppressor protein frequently inactivated in schwannoma, meningioma, and malignant mesothelioma (MM). The sequence of merlin is similar to that of ezrin/radixin/moesin (ERM) proteins which crosslink actin with the plasma membrane, suggesting that merlin plays a role in transducing extracellular signals to the actin cytoskeleton. Merlin adopts a distinct closed conformation defined by specific intramolecular interactions and regulates diverse cellular events such as transcription, translation, ubiquitination, and miRNA biosynthesis, many of which are mediated through Hippo and mTOR signaling, which are known to be closely involved in cancer development. MM is a very aggressive tumor associated with asbestos exposure, and genetic alterations in *NF2* that abrogate merlin’s functional activity are found in about 40% of MMs, indicating the importance of *NF2* inactivation in MM development and progression. In this review, we summarize the current knowledge of molecular events triggered by *NF2*/merlin inactivation, which lead to the development of mesothelioma and other cancers, and discuss potential therapeutic targets in merlin-deficient mesotheliomas.

## 1. Introduction

Mutations in the neurofibromatosis type 2 (*NF2*) gene are responsible for neurofibromatosis 2, a dominantly inherited familial cancer syndrome characterized by the formation of bilateral vestibular schwannomas and meningiomas [1,2]. Besides sporadic schwannomas [3] and meningiomas [4], frequent biallelic inactivation of *NF2* was also found in malignant mesothelioma (MM), a very aggressive tumor which is not associated with the *NF2* cancer syndrome [5,6]. Tumors carrying *NF2* mutations are also observed, albeit infrequently, in multiple organs such as the breast, the prostate, the liver, and the kidney [7,8], indicating a significant role of *NF2* in the development of various human malignancies.

Findings in mouse models support the biological function of *NF2* as a tumor suppressor gene. Since it was shown that a homozygous mutation in the *NF2* gene of mice causes embryonic death by day 6.5 of their development [9], the role of *NF2* as a tumor suppressor gene has been studied in mice that are heterozygous for *NF2* mutations. It was found to develop a variety of malignant tumors, including lymphoma, sarcoma, and carcinoma [10,11]. Furthermore, some studies revealed the involvement of *NF2* in the development of malignant plural mesothelioma after asbestos exposure. Thus, heterozygous *NF2*^+/−^ mice had a higher sensitivity to asbestos, which resulted in an increased risk of malignant mesothelioma formation compared to wild-type *NF2*^+/+^ mice [2,12]. A direct injection of the Adeno-Cre virus into the pleural cavity of adult mice resulted in a conditional knockout of oncosuppressor genes, which further demonstrated that the loss of *NF2*, together with *Tp53* or *Ink4a*/*Arf*, frequently causes the development of mesothelioma which closely mimicked human MM [13]. It was also shown that the restoration of *NF2* expression in *NF2*-deficient mesothelioma cells significantly inhibited their growth [14,15,16]. These in vitro and in vivo data strongly support the role of *NF2* inactivation in mesothelioma development.

## 2. Domain Organization and Functions of Merlin

### 2.1. NF2 Transcript Variants

**Figure 1:**
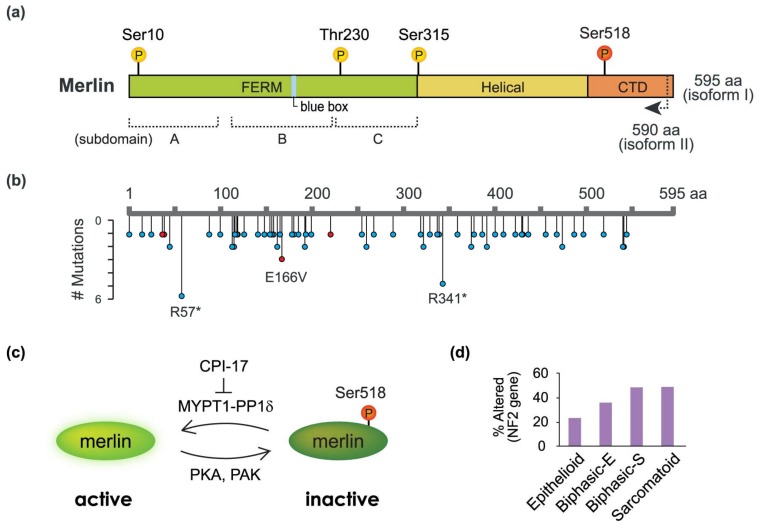
Mechanisms underlying the activation/inactivation of merlin. (**a**) Domain organization of merlin. The protein consists of the N-terminal FERM (band 4.1/ezrin/radixin/moesin) domain (green) comprising three subdomains (A, B, and C), a central helical domain (yellow), and a C-terminal domain (CTD, orange). Major phosphorylation sites are indicated; (**b**) *NF2* mutations and their frequency in pleural and peritoneal cancers. Nonsense/frameshift (blue) and missense (red) mutations registered in COSMIC (Catalogue of Somatic Mutations in Cancer; http://cancer.sanger.ac.uk/cosmic/) as of 27 February 2018, are mapped; (**c**) Phosphorylation-dependent inactivation of merlin. Phosphorylation at Ser518 inactivates merlin and inhibits its growth suppression activity; (**d**) Frequency of genetic alterations in the *NF2* gene, including mutations, fusions, and copy number variations in different subtypes of malignant pleural mesothelioma based on an analysis of 211 malignant plural mesothelioma samples. The data were adapted from Bueno et al. [24].

The *NF2* gene is located in the chromosomal region 22q12 [1,17]; the gene contains 17 exons and spans approximately 95 kb of DNA. *NF2* transcripts undergo alternative splicing, thereby generating multiple isoforms [18], and variable *NF2* transcripts are observed in human mesotheliomas [5,12]. Two transcripts, one lacking exon 16 and the other containing all 17 exons, are the predominant variants encoding isoforms I and II; the first contains 595 amino acids, while the second, which is generated by the insertion of exon 16 into mRNA which creates a new stop codon, contains 590 amino acids and is identical to isoform I in the first 579 residues (Figure 1A). Initially, it was thought that isoform II lacked anticancer activity [19,20]; however, later studies showed that both isoforms exhibited the function of tumor suppression [21,22,23].

### 2.2. Domain Organization

The *NF2* gene product, named merlin, is widely expressed in various human tissues and is most closely related to the ezrin/radixin/moesin (ERM) family proteins, which are localized at cell-surface structures such as ruffling membranes and cell–cell adhesion sites, and connect actin filaments to the plasma membrane. The significant similarity in amino acid sequences between merlin and ERM proteins suggests that merlin can be associated with the actin cytoskeleton and the organization of membrane domains [25].

A structural analysis shows that merlin consists of three domains: the N-terminal FERM (band 4.1, ezrin, radixin, moesin) domain containing three subdomains (A, B, and C), the central helical domain, and the C-terminal domain (CTD) (Figure 1A). Merlin shares 45–47% sequence similarity with the ERM family members, especially in the conserved FERM domain (60–70%). The FERM of merlin binds to membrane proteins such as hyaluronate receptor CD44 [26,27], adaptor molecule Na^+^/H^+^ exchanger three, regulating factor one (NHERF/EBP50) [28,29], and E-cadherin [30]. Furthermore, the FERM mediates protein binding to phospholipids such as phosphatidylinositol 4,5-bisphosphate (PIP2) [31,32]. Despite the similarity in the binding properties between merlin and ERM proteins, their CTDs show distinct binding preferences. The CTDs of ERM proteins have actin-binding sites [33] linking the plasma membrane to the actin cytoskeleton, whereas merlin lacks the region corresponding to the C-terminal F-actin-binding site [34] and interacts with actin fibers through residues 1–27 and 280–323, which seem to be sufficient for the binding [35]. Moreover, merlin has a unique seven-amino-acid stretch (residues 177–183) in the FERM domain, named the ‘blue box’, which is conserved from fly to mammalian proteins but is lacking in ERM family members [34,36]. Alanine substitution in, or deletion of, this region produces unique merlin mutants, which have dominant-negative activity and result in an excessive proliferation of wing epithelial cells in flies [36] and a loss of contact inhibition in mammalian cells [26,37]. The unique characteristics of merlin domains suggest that the regulation of merlin is distinct from that of ERM proteins.

### 2.3. Molecular Conformation and Phosphorylation

ERM proteins have a ‘closed’ inactive conformation formed by the binding of CTD to the N-terminal FERM, whereas the phosphorylation of C-terminal residues disrupts the interaction, resulting in the ‘open’ active state, where the released FERM and CTD can bind to cell adhesion molecules and actin filaments, respectively [31]. Although a C-terminal phosphorylation site, threonine 576, critical for the conformational change in ERM proteins, is also conserved in merlin; the Thr576Ala substitution does not affect merlin’s ability to suppress cell growth and motility [38]. Aside from this, the phosphorylation of merlin at serine 518 abrogates its growth inhibition activity [38,39]. These findings indicate that in merlin, phosphorylation causes inactivation, which is in contrast to its effect in ERM proteins (Figure 1C).

Merlin phosphorylation at Ser518 was frequently observed in mesothelioma cells expressing full-length merlin [12]. Moreover, CPI-17, a cellular inhibitor of myosin phosphatase targeting subunit 1 (MYPT1-PP1δ), was increased in mesothelioma cells with full-length *NF2* compared to normal pleura or mesothelioma with truncated *NF2* [38]. As MYPT1-PP1δ dephosphorylates merlin at Ser518 [40], CPI-17 upregulation would result in an increased phosphorylation and inactivation of merlin (Figure 1C). These findings suggest that merlin can be inactivated not only by mutations but also through posttranslational modifications occurring in mesothelioma cells.

The Ser518 phosphorylation in merlin is independently catalyzed by distinct protein kinases such as p21-activated kinase (PAK) [41,42,43] and protein kinase A (PKA) [44]. PAK causes a phosphorylation-dependent inactivation of merlin and promotes the loss of contact inhibition of proliferation [45], whereas PKA, in addition to Ser518, also phosphorylates Ser10 that is not conserved in ERM proteins, which results in increased cell migration [46]. Another protein kinase, AKT, phosphorylates merlin at Thr230 and Ser315, which appears to stimulate ubiquitin-dependent protein degradation [47].

Given the data on ERM proteins, Ser518 phosphorylation in merlin has been suggested to change its conformation from a ‘closed’ to an ‘open’ state [48]. Although the FERM and the CTD of merlin bind each other, their mutual affinity is low compared to that in ERM proteins [49], suggesting that merlin may not form a fully closed form. Instead, phosphorylation was shown to rather strengthen the head-to-tail folding in merlin [23]. Analysis by fluorescence resonance energy transfer (FRET) suggests that phosphorylation causes a subtle conformation change in merlin [50]. Furthermore, although merlin isoform II does not form the ‘closed’ state since it lacks five C-terminal residues [51], both isoforms I and II exhibit antitumor activity [21,22,23]. Cumulatively, these findings suggest that the phosphorylation at Ser518 would inactivate merlin without the accompanying dynamic conformational change observed in ERM proteins.

### 2.4. NF2 Inactivation in Mesothelioma

In addition to a frequent loss of the 22q12 region, which is the locus of the *NF2* gene, mutations within the entire *NF2* coding region are common for mesothelioma (Figure 1B). Nonsense mutations either totally abolish merlin expression or lead to the production of truncated forms. The functional activity of the truncated merlin variants, especially those with a short deletion at the C-terminus, has not been fully characterized. However, it was shown that the mutant with a C-terminal deletion of 40 residues was incapable of restoring proper growth inhibition in *NF2*-null mesothelioma cells [15], and that merlin truncated by 63 residues at the C-terminus did not cause growth arrest of primary Schwann cells [13], indicating the importance of the CTD for the antitumor activity of merlin. Therefore, nonsense mutations in *NF2*, even those occurring close to the C-terminus, are suggested to produce functional defects and are responsible for mesothelioma development. In contrast, the impact of missense mutations that cause amino acid substitutions is less understood, and it is unclear as to how and to what extent individual mutations affect merlin tumor-suppressive function. Although the pathogenic activity of several missense mutants identified in tumors have been studied [14], further investigation is required for a complete understanding of the effect produced by merlin mutations on tumor progression. In addition, *NF2* gene rearrangements are also frequently detected in MM, and each *NF2* gene fusion variant was thought to cause functional inactivation [24].

Regarding gene mutation frequency in MM, gene alterations in *NF2* are considered to be the second most common after those in BAP1. Developed mesothelioma tumors have different histological subtypes: epithelioid, sarcomatoid, and biphasic MMs. An expression analysis of 211 malignant plural mesothelioma samples suggested that among the subtypes, sarcomatoid tumors had the highest *NF2* mutation rate, while epithelioid tumors had the lowest *NF2* mutation rate [24] (Figure 1D). Furthermore, hemizygous *NF2* loss has been shown to decrease both the overall survival and the progression-free survival in a cohort of 86 peritoneal mesothelioma patients [52]. These data suggest that *NF2* inactivation might be involved in the epithelial–mesenchymal transition during metastasis, and that the development of sarcomatoid mesotheliomas is characterized by a poorer overall survival compared to the epithelioid subtype.

### 2.5. Loss of Contact Inhibition in NF2-Deficient Cells

**Figure 2:**
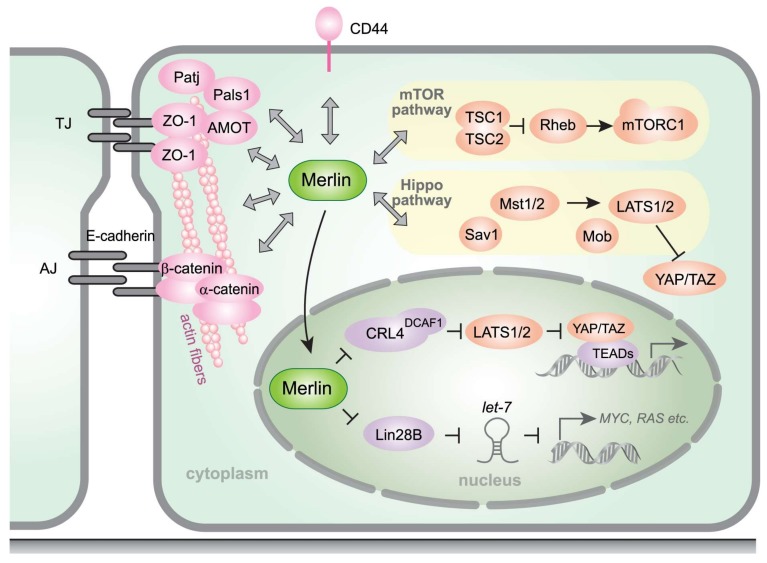
A model of the *NF2*/merlin signaling pathway. Merlin is involved in contact inhibition by interacting with many membrane-associated proteins such as CD44 [26,27], the angiomotin (AMOT) –Patj–Pals1 complex [48], E-cadherin–α-catenin [30,55], and actin fibers. A loss of merlin expression disrupts cancer-related signaling through the Hippo and mTOR pathways. Merlin is also localized in the nucleus where it binds to and inhibits E3 ubiquitin ligase CRL4^DCAF1^, which promotes LATS1/2 degradation [66,67], and RNA-binding protein Lin28B, which suppresses let-7 miRNAs that are involved in the silencing of oncogenes such as *MYC* and *RAS* [68]. TJ: tight junction; AJ: adherens junction; ZO-1: Zonula occludens-1; AMOT: angiomotin; mTOR: mechanistic target of rapamycin; TSC1/2: tuberous sclerosis complex 1/2; Rheb: Ras homolog enriched in brain; Sav1: Salvador Family WW Domain Containing Protein 1; Mst1/2: mammalian Ste20-like kinase 1/2; Mob: Mps one binder kinase activator-like protein; YAP: yes-associated protein 1; TAZ: WW domain-containing transcription regulator 1; CRL4: Cullin-RING ubiquitin ligase 4; DCAF1: DDB1- and CUL4-associated factor 1; LATS1/2: large tumor suppressor kinase 1/2; TEAD: TEA domain transcription factor; Lin28B: lin-28 homolog B.

Contact inhibition, a regulatory mechanism providing cell growth arrest at confluence in tissue culture, is frequently disrupted in cancer cells [53], and *NF2*-null cells grow to a significantly higher density compared to wild-type cells, suggesting that *NF2* controls tumor progression. The mechanism underlying the merlin regulation of growth arrest in response to cell confluence has been addressed in several studies. For example, it has been shown that merlin forms a complex with CD44, which is activated by the stimulation of extracellular hyaluronate, resulting in growth inhibition of rat schwannoma cells in vitro. Other studies have suggested that merlin regulates contact inhibition through small GTPase Rac1 [45,54], α-catenin, cell-polarity protein Par3 [55], and a tight-junction-associated complex composed of angiomotin (AMOT), Patj, and Pals1 [48] (Figure 2, shown in pink). These findings suggest that merlin could sense its environmental conditions and control cell growth via complex interactions with signaling proteins involved in cell–cell adhesion.

### 2.6. Subcellular Localization

FERM domain-containing proteins link plasma membrane receptors to cytoskeleton components [12]. Consistent with this notion, immunostaining with merlin-specific antibodies detects merlin at the cell membrane or the ruffling edges in human fibroblasts, meningioma cells, and Schwann cells [56,57]. Although the localization of the wild-type or the mutant merlin in mesothelial and mesothelioma cells is not defined, we have observed exogenously expressed full-length V5-tagged merlin both at the plasma membrane and in the cytoplasm of merlin-negative mesothelioma cells [58]. However, as merlin localization is dynamically regulated in response to various signals (described below), a further detailed investigation is necessary.

## 3. Proteins and Signaling Related to Merlin’s Functions

### 3.1. Hippo Signaling Pathway

Merlin exerts its tumor-suppressive effects by controlling the expression of oncogenic genes through the activation of Hippo signaling (Figure 2, shown in orange). The Hippo pathway is composed of core proteins including MST1/2 (Mammalian STE20-Like Protein Kinases), SAV1 (Salvador Family WW Domain Containing Protein 1), MOB (MOB Kinase Activators), and LATS1/2 (Large Tumor Suppressor Kinase 1/2) [59]. At the plasma membrane, merlin recruits LATS1/2 kinases which directly phosphorylate the downstream effectors of the Hippo pathway, YAP (Yes-Associated Protein) and its paralogue TAZ (WW Domain-Containing Transcription Regulator 1, alternatively WWTR1), thus preventing their translocation to the nucleus and inhibiting their function as transcription co-activators. Alternatively, Hippo pathway inactivation induces an accumulation of underphosphorylated YAP and TAZ in the nucleus and their association with DNA-binding TEAD (TEA Domain Transcription Factor) family proteins, which upregulates the transcription of multiple oncogenic genes [60]. Along with *NF2* mutations, gene alterations are also frequently observed in Hippo pathway components, including LATS1/2, SAV1, and LIM-domain containing protein AJUBA, a *Drosophila* djub homolog and LATS1/2 binding partner [61,62]. High-level amplification of the 11q22 locus encompassing the *YAP* gene was also observed in a small subset of MMs [58]. These results indicate that the disruption of Hippo signaling plays a central role in the transformation of mesothelial cells.

YAP activation in mesothelial cells drastically changes their behavior. Kakiuchi et al. [63] have shown that the expression of constitutively active YAP Ser127Ala mutants in immortalized mesothelial cells promotes their growth in vitro, as well as tumor formation after their transplantation in mice. Conversely, YAP knockdown inhibits cell growth, motility, and invasion in mesothelioma cells with activated YAP, but did not show any effects in cells without YAP activation [64]. Furthermore, these studies showed that the YAP-dependent transcriptional activations of cyclin D2 (CCND2), forkhead box M1 (FOXM1), and phospholipase C beta 4 (PLCB4) are involved in mesothelioma cell growth [63,64], suggesting that activated YAP influences diverse cellular processes, thereby resulting in mesothelial cell transformation. The role of TAZ in mesothelioma has not been defined yet, but considering its functional redundancy with YAP, the oncogenic function of TAZ could be predicted.

### 3.2. DCAF1

It has been reported that merlin can translocate into the nucleus, where it binds to DCAF1 (also known as VprBP) through the N-terminal FERM domain [65]. DCAF1 is a substrate adaptor of E3 ubiquitin ligase CRL4^DCAF1^ containing CUL4 and DDB1. The interaction between merlin and DCAF1 depends on merlin activation, since neither the Ser518Asp phosphomimetic mutant, nor the Ser64Ala mutant, which lacks tumor-suppressor activity, bind to CRL4^DCAF1^. Merlin inhibits the activity of CRL4^DCAF1^, which regulates ubiquitination of target proteins. It was shown that LATS1/2 are functional targets of CRL4^DCAF1^ and that in tumors with mutated *NF2*, such as mesothelioma, activated CRL4 induces LATS1/2 ubiquitination to promote their degradation and YAP/TAZ activation, thus stimulating oncogenesis [66] (Figure 2, shown in purple). These results suggest that DCAF1 and CRL4^DCAF1^ are potential therapeutic targets for merlin-deficient mesothelioma. Cooper et al. [67] tested whether CRL4^DCAF1^ inhibition with NEDD8-activating enzyme (NAE) inhibitor MLN4924 could suppress the growth of tumor cells carrying *NF2* mutations. MLN4924 alone caused only a moderate inhibition of mesothelioma cell growth, but the combination of MLN4924 and GDC-0980, an mechanistic target of rapamycin/phosphatidylinositol 3-kinase (mTOR/PI3K) inhibitor, strongly suppressed cell proliferation. Despite blocking a broad spectrum of Cullin–RING E3 ligases including CRL4^DCAF1^, NF2–NAE inhibitors could be a promising target for therapeutic intervention in patients with merlin-negative mesothelioma.

### 3.3. PI3K/AKT/mTOR Signaling Pathway

mTOR is a serine/threonine kinase that plays a key role in cell growth and proliferation. The mTOR signaling pathway has been reported to be frequently activated in a variety of human malignancies, indicating its close involvement in carcinogenesis.

mTOR is composed of two distinct complexes, mTOR complex 1 (mTORC1) and mTOR complex 2 (mTORC2) [69,70]; both of them contain mTOR kinase and a mTORC subunit mLST8, which is suggested to stabilize the structure of the mTOR catalytic domain [71]. mTORC1 binds to raptor, whereas mTORC2 binds to rictor and Sin1, forming functional kinase complexes. mTORC1 and its activator Rheb have been shown to enhance protein translation and pyrimidine nucleotide biosynthesis, thereby promoting cell growth and proliferation [72,73,74].

The involvement of the mTOR pathway in mesothelioma formation has been suggested in several studies. Thus, López-Lago et al. [75] showed, using a panel of malignant mesothelioma cell lines, that the loss of merlin correlated with the activation of mTORC1 signaling and the sensitivity to rapamycin. Similarly, James et al. [76] reported that merlin-deficient meningioma cells also exhibited constitutive mTORC1 activation and increased growth. Furthermore, it was demonstrated that the concurrent loss of Tp53 and tuberous sclerosis 1 (TSC1), a negative regulator of Rheb–mTORC1 signaling, induces the development of peritoneal mesothelioma in mice [77]. Immunohistochemical analysis of human mesotheliomas revealed the hyperactivation of mTORC1 and the reduced expression of TSC2, which binds to TSC1 and negatively regulates the activation of mTORC1 by Rheb. These findings suggest that mTOR activation caused by merlin inactivation plays a significant role in mesothelioma development (Figure 2, shown in orange).

The deregulation of mTORC1 signaling in mesothelioma cells can be attributed to changes in the state of various upstream effectors. AKT, an mTORC1 activator and mTORC2 substrate, is stimulated in more than 60% of malignant mesothelioma cell lines and tumors [78,79]; furthermore, the homozygous deletion of PTEN, a negative regulator of AKT signaling, has also been reported in mesothelioma cells [78,79]. PTEN loss leads to an increase in phosphatidylinositol (3,4,5)-triphosphate (PIP3), resulting in the activation of both mTORC1 and mTORC2 signaling. These data suggest that the activation of mTORC1, as well as mTORC2, may be involved in mesothelioma development. On the other hand, no activating mutations in the *MTOR*, nor the *RHEB* genes, have been identified in mesothelioma cells to date, although such mutations were shown to cause the hyper-activation of mTORC1 [80,81] observed in mesothelioma. The biological role of mTORC1 in mesothelioma formation is now beginning to be examined.

### 3.4. Lin28B and let-7 miRNAs

An RNA-binding protein, Lin28B, has been recently reported to be an alternative binding partner of merlin. Lin28B is involved in cell growth and reprogramming [82,83] and suppresses the biogenesis of the let-7 microRNAs (miRNAs) that function as tumor suppressors by silencing the expression of several oncogenes such as *MYC* and *RAS* [84,85]. Hikasa et al. [68] found that merlin bound to Lin28B through the FERM domain and translocated Lin28B from the nucleus to cytoplasm, leading to let-7 miRNA maturation (Figure 2, shown in purple). The association between merlin and Lin28B is induced when merlin is dephosphorylated, which occurs at high cell density, suggesting a novel mechanism in which merlin exerts cell-density-dependent tumor suppression through let-7 miRNA maturation.

### 3.5. TRAF7

Recurrent mutations in the *TRAF7* gene are observed in mesothelioma cells. *TRAF7* belongs to tumor necrosis factor (TNF) receptor-associated factors (TRAFs) possessing E3 ubiquitin ligase activity [86], and it was shown to promote ubiquitination of an apoptosis inhibitor, FLIP [87], which is increased in mesothelioma cells [88]. FLIP inhibition by small interfering RNA (siRNA) sensitizes mesothelioma cells to Fas- and TRAIL-induced apoptosis, suggesting a role of FLIP in protecting cells from death signals. Interestingly, *TRAF7* and *NF2* mutations are mutually exclusive in malignant pleural mesothelioma [24] as well as in meningioma [89], suggesting that merlin and *TRAF7* may use a common signal transduction pathway.

## 4. Potential Molecular Targets in Merlin-Negative Mesothelioma

### 4.1. FAK Inhibitors

Focal adhesion kinase (FAK) is a serine/threonine kinase that mediates signals from focal adhesion complexes to the cell growth and migration machinery. FAK is elevated in most human cancers, and its inhibition has been recognized as a novel approach to targeted anticancer therapy against various types of solid tumors. In 2014, Shapiro and colleagues [90] reported that a beneficial effect of an FAK inhibitor, VS-4718 (alternatively PND-1186), on MM cells lacking merlin expression was the increased sensitivity of MM cells to VS-4718 in vitro and in tumor xenograft models. Therefore, FAK inhibitors were considered as potential candidates for mesothelioma therapy. However, a phase II clinical trial investigating the effects of an FAK inhibitor, defactinib (VS-6063), on merlin-deficient mesotheliomas was terminated early due to its futility, and the reason for the poor clinical performance is currently unclear. A recent study on the pharmacological effects of FAK inhibitors has demonstrated a significant correlation between *E*-cadherin mRNA levels and VS-4718 in merlin-negative mesothelioma [15], suggesting that E-cadherin may serve as a promising biomarker for predicting the response to FAK inhibitors in mesothelioma, which should be tested in clinical settings.

### 4.2. YAP Inhibitors

The screening of more than 3300 Food and Drug Administration (FDA)-approved small molecules resulted in the identification of verteporfin as a novel compound that disrupts the YAP–TEAD interaction and inhibits YAP oncogenic activity [91]. Verteporfin, a benzoporphyrin derivative, is currently used in clinics as a photosensitizer in photodynamic therapy for macular degeneration. The compound is activated by 690 nm far-red light, generating reactive oxygen species (ROS) which eliminate abnormal blood vessels; however, its inhibition of YAP–TEAD interactions does not require light activation. Several in vitro studies revealed that verteporfin can suppress the growth, the migration, and the tumorsphere formation of cultured MM cells [92,93]. Recently, CIL56 (also named CA3), a small molecule that induces cellular ferroptosis through ROS production [94], has been identified as a novel YAP inhibitor. By preventing the interaction between YAP and TEAD, CIL56 strongly inhibited esophageal adenocarcinoma cell growth both in vitro and in vivo [95]. Therefore, YAP inhibitors may be effective anticancer drugs for mesothelioma and other tumors in which YAP/TAZ are activated through the disruption of the Hippo pathway.

In addition to the nucleocytoplasmic shuttling of YAP/TAZ, a recent study revealed a mechanism for the intracellular translocation of TEAD proteins. The findings of Lin et al. [96] suggest that environmental stresses such as osmotic stress, high cell density, and cell detachment promote TEAD translocation from the nucleus to the cytoplasm via p38 mitogen-activated protein kinase (MAPK). Interestingly, TEAD nucleocytoplasmic transfer occurred in a Hippo-independent manner and suppressed YAP and YAP-dependent cancer cell growth, suggesting that the regulation of TEAD translocation might serve as another therapeutic strategy in merlin-deficient tumors.

### 4.3. mTOR Inhibitors

Given the emerging role of mTOR in mesothelioma development and proliferation, mTOR inhibitors are thought to be promising drugs against merlin-negative mesothelioma. Unfortunately, however, a phase II clinical trial of everolimus, a first-generation mTOR inhibitor rapamycin analog (so-called rapalog), demonstrated that there was insufficient activity in patients with advanced mesothelioma [97]. The reason for this limited success is not fully understood; it is possible that certain rapamycin-resistant functions of mTORC1 [98] may account for the low efficacy of rapalogs.

To date, various types of improved mTOR inhibitors have been developed. Second-generation mTOR inhibitors (also called ATP-competitive mTOR inhibitors) directly compete with ATP for the binding to the mTOR kinase domain, thus completely inhibiting both mTORC1 and mTORC2. PI3K/mTOR dual inhibitors also target the ATP-binding pocket; the advantage of these compounds is that they recognize the ATP-binding site not only in mTOR but also in PI3K. Considering the reports of mTORC2 activation in mesothelioma cells due to the loss of PTEN, which is an mTORC2 negative regulator, or the increased phosphorylation of mTORC2 substrate AKT [79,99], PI3K/mTOR dual inhibitors are predicted to be more effective in suppressing mesothelioma growth than rapalogs. Moreover, a combination treatment with mTOR or PI3K/mTOR inhibitors together with other antitumor drugs appears to be a reasonable approach, because mTOR signaling is involved in a compensatory pathway that renders cancer cells drug resistant; thus, increased mTORC1 activity in breast and pancreatic cancer cells confers resistance to cyclin-dependent kinase 4/6 (CDK4/6) inhibitors [100,101]. Although in vivo experiments are lacking, in vitro data indicate that a combination treatment with the CDK4/6 inhibitor, palbociclib, and a PI3K/mTOR dual inhibitor exerts a synergistic effect on mesothelioma cell growth [102].

Recently, a third-generation mTOR inhibitor which overcomes the resistance to first- and second-generation mTOR inhibitors has been developed [103] and already showed promise by exhibiting a higher efficacy in glioblastomas compared to previous mTOR inhibitors [104]. Although the antitumor activity of the new mTOR inhibitors against mesothelioma has yet to be demonstrated, enhanced clinical benefits can be expected.

### 4.4. Statins

Statins are inhibitors of 3-hydroxyl-3-methyl coenzyme A (HMG–CoA) reductase, the rate-limiting enzyme of the mevalonate pathway for the biosynthesis of mevalonate and downstream isoprenoids, which are suggested to have beneficial effects on several cancers, including colorectal cancer, breast cancer, and melanoma [105]. The therapeutic potential of statins for suppressing mesothelioma cell growth has been reported in vitro and in mouse xenografts [106,107]. Furthermore, statins are suggested to have synergistic or additive antitumor effects when used with other drugs [108,109,110]. Recently, it was reported that mesothelioma cells with *NF2* and/or *LATS2* mutations were more sensitive to fluvastatin compared to those with *BAP1* mutations [111], whereas merlin-negative breast cancer cells showed sensitivity to simvastatin [112]. The regulation of YAP and TAZ through the mevalonate pathway [113] suggests that statins may show a more significant effect on cell growth in *NF2*-deficient mesothelioma and other types of tumors.

### 4.5. COX2 Inhibitors

It has been demonstrated that YAP activation in *NF2*-null Schwann cells promotes the transcription of the *PTGS2* gene encoding cyclooxygenase 2 (COX-2), the key enzyme in prostaglandin biosynthesis. Interestingly, the treatment of NF2-null Schwann or schwannoma cells with a COX-2 inhibitor, celecoxib, dramatically inhibited cell growth in vitro and in vivo [114], which suggests that COX-2 is a potential therapeutic target in *NF2*-null tumors. However, a recent study showed that celecoxib failed to prevent the generation of schwannomas in a genetically engineered mouse model of *NF2* inactivation, although COX-2 expression was increased in tumors that developed in these mice [115]. Considering the controversial results on COX-2 as a target in *NF2*-inactive tumors, further investigations are required in this direction.

## 5. Conclusions

Malignant mesothelioma is highly refractory to conventional therapies, and the current chemotherapeutic approach approved in clinics is still based on a combination of platinum and an antifolate, pemetrexed [116]. *NF2* is one of the most frequently mutated genes in mesothelioma; therefore, the restoration of *NF2* functions is expected to cure a large population of mesothelioma patients. However, the introduction of tumor suppressor genes in every tumor cell and the subsequent expression of the encoded proteins at levels that are comparable to those of normal cells remain highly challenging [117]. Growing evidence demonstrates that merlin is distributed in multiple subcellular compartments and suppresses a number of proteins and signaling pathways that are related to tumor progression [118]. Once *NF2* is inactivated, these oncogenic mechanisms are constitutively induced, conferring malignant phenotypes to the cells; therefore, targeting merlin-dependent molecular pathways is a promising strategy for the treatment of *NF2*-deficient cancers. The restoration of the Hippo signaling and the inhibition of the PI3K/AKT/mTOR pathway are predicted to exert potent anticancer effects, but the clinical performance of the perspective drugs has not yet been evaluated, and the mechanisms underlying *NF2* control of these signaling pathways in mesothelial and other cells are still unknown.

Although *NF2* is frequently inactivated in MMs, recent progress in *NF2*-targeted therapies has been limited [119]. To search for more effective drugs against *NF2*-deficient mesothelioma cells, we have to understand when, where, and how merlin exerts its tumor-suppressive effects, especially in mesothelial cells. Further, the roles of downstream signals that are activated by *NF2* loss in mesothelioma progression also remain incompletely defined. For example, is the activation of YAP via the inactivation of the Hippo signaling pathway enough for mesothelioma formation? If so, why are *YAP* gene mutations that constitutively activate their transcription activity undetected in MMs? The activation of *TAZ* in merlin-deficient MM cells should be evaluated as a potential key oncogene that drives tumor initiation and progression together with YAP. It is to be noted that *NF2* loss might be involved in drug resistance. A genome-wide CRISPR screen in human cells has identified *NF2* as the highest-ranking candidate whose loss is involved in the resistance to vemurafenib, a therapeutic RAF inhibitor [120]. Future studies focusing on defining the alteration of molecular networks caused by the loss of merlin expression would further foster the development of new therapeutic strategies in mesothelioma.

## Abbreviations

AMOT	angiomotin
BAP1	BRCA1 Associated Protein 1
CCND2	cyclin D2
CDK	cyclin-dependent kinase
COX-2	cyclooxygenase 2
CPI-17	protein kinase C-potentiated inhibitor protein of 17 kDa
CRL4	Cullin-RING ubiquitin ligase 4
CTD	C-terminal domain
CUL4	Cullin 4
DCAF1	DDB1- and CUL4-associated factor 1
DDB1	damaged DNA binding protein 1
ERM	ezrin/radixin/moesin
FAK	focal adhesion kinase
FDA	Food and Drug Administration
FERM	band 4.1/ezrin/radixin/moesin
FLIP	caspase-like apoptosis regulatory protein
FOXM1	forkhead box M1
LATS1/2	large tumor suppressor kinase 1/2
Lin28B	lin-28 homolog B
MAPK	mitogen-activated protein kinase
mLST8	mammalian lethal with Sec13 protein 8
MM	malignant mesothelioma
Mob	Mps one binder kinase activator-like protein
mTOR	mechanistic target of rapamycin
mTORC	mTOR complex
MST1/2	mammalian Ste20-like protein kinase 1/2
MYPT1-PP1δ	myosin phosphatase target subunit 1-protein phosphatase 1 δ
NAE	NEDD8-activating enzyme
NF2	neurofibromatosis type 2
PAK	p21-activated kinase
Pals1	protein associated with Lin7-1
Patj	Pals1-associated tight junction protein
PI3K	phosphatidylinositol 3-kinase
PKA	protein kinase A
PLCB4	phospholipase C beta 4
PTEN	phosphatase and tensin homolog
Rheb	Ras homolog enriched in brain
ROS	reactive oxygen species
SAV1	Salvador Family WW Domain Containing Protein 1
siRNA	small interfering RNA
TAZ	WW domain-containing transcription regulator 1
TEAD	TEA domain transcription factor
TNF	tumor necrosis factor
TRAF	TNF receptor-associated factor
TRAIL	TNF-related apoptosis inducing ligand
TSC1/2	tuberous sclerosis complex subunit 1/2
VprBP	viral protein R (VPR)-binding protein
YAP	Yes-associated protein 1
ZO-1	Zonula occludens-1

## References

[1] Trofatter J.A., MacCollin M.M., Rutter J.L., Murrell J.R., Duyao M.P., Parry D.M., Eldridge R., Kley N., Menon A.G., Pulaski K. (1993). A novel moesin-, ezrin-, radixin-like gene is a candidate for the neurofibromatosis 2 tumor suppressor. Cell.

[2] Altomare D.A., Vaslet C.A., Skele K.L., De Rienzo A., Devarajan K., Jhanwar S.C., McClatchey A.I., Kane A.B., Testa J.R. (2005). A mouse model recapitulating molecular features of human mesothelioma. Cancer Res..

[3] Stemmer-Rachamimov A.O., Xu L., Gonzalez-Agosti C., Burwick J.A., Pinney D., Beauchamp R., Jacoby L.B., Gusella J.F., Ramesh V., Louis D.N. (1997). Universal absence of merlin, but not other ERM family members, in schwannomas. Am. J. Pathol..

[4] Ruttledge M.H., Sarrazin J., Rangaratnam S., Phelan C.M., Twist E., Merel P., Delattre O., Thomas G., Nordenskjold M., Collins V.P. (1994). Evidence for the complete inactivation of the *NF2* gene in the majority of sporadic meningiomas. Nat. Genet..

[5] Sekido Y., Pass H.I., Bader S., Mew D.J., Christman M.F., Gazdar A.F., Minna J.D. (1995). Neurofibromatosis type 2 (*NF2*) gene is somatically mutated in mesothelioma but not in lung cancer. Cancer Res..

[6] Bianchi A.B., Mitsunaga S.I., Cheng J.Q., Klein W.M., Jhanwar S.C., Seizinger B., Kley N., Klein-Szanto A.J., Testa J.R. (1995). High frequency of inactivating mutations in the neurofibromatosis type 2 gene (*NF2*) in primary malignant mesotheliomas. Proc. Natl. Acad. Sci. USA.

[7] Li W., Cooper J., Karajannis M.A., Giancotti F.G. (2012). Merlin: A tumour suppressor with functions at the cell cortex and in the nucleus. EMBO Rep..

[8] Petrilli A.M., Fernandez-Valle C. (2016). Role of Merlin/NF2 inactivation in tumor biology. Oncogene.

[9] McClatchey A.I., Saotome I., Ramesh V., Gusella J.F., Jacks T. (1997). The *NF2* tumor suppressor gene product is essential for extraembryonic development immediately prior to gastrulation. Genes Dev..

[10] Giovannini M., Robanus-Maandag E., van der Valk M., Niwa-Kawakita M., Abramowski V., Goutebroze L., Woodruff J.M., Berns A., Thomas G. (2000). Conditional biallelic NF2 mutation in the mouse promotes manifestations of human neurofibromatosis type 2. Genes Dev..

[11] McClatchey A.I., Saotome I., Mercer K., Crowley D., Gusella J.F., Bronson R.T., Jacks T. (1998). Mice heterozygous for a mutation at the *NF2* tumor suppressor locus develop a range of highly metastatic tumors. Genes Dev..

[12] Thurneysen C., Opitz I., Kurtz S., Weder W., Stahel R.A., Felley-Bosco E. (2009). Functional inactivation of NF2/merlin in human mesothelioma. Lung Cancer.

[13] Jongsma J., van Montfort E., Vooijs M., Zevenhoven J., Krimpenfort P., van der Valk M., van de Vijver M., Berns A. (2008). A conditional mouse model for malignant mesothelioma. Cancer Cell.

[14] Poulikakos P.I., Xiao G.H., Gallagher R., Jablonski S., Jhanwar S.C., Testa J.R. (2006). Re-expression of the tumor suppressor NF2/merlin inhibits invasiveness in mesothelioma cells and negatively regulates FAK. Oncogene.

[15] Kato T., Sato T., Yokoi K., Sekido Y. (2017). *E*-cadherin expression is correlated with focal adhesion kinase inhibitor resistance in Merlin-negative malignant mesothelioma cells. Oncogene.

[16] Xiao G.H., Gallagher R., Shetler J., Skele K., Altomare D.A., Pestell R.G., Jhanwar S., Testa J.R. (2005). The *NF2* tumor suppressor gene product, merlin, inhibits cell proliferation and cell cycle progression by repressing cyclin D1 expression. Mol. Cell. Biol..

[17] Rouleau G.A., Merel P., Lutchman M., Sanson M., Zucman J., Marineau C., Hoang-Xuan K., Demczuk S., Desmaze C., Plougastel B. (1993). Alteration in a new gene encoding a putative membrane-organizing protein causes neuro-fibromatosis type 2. Nature.

[18] Chang L.S., Akhmametyeva E.M., Wu Y., Zhu L., Welling D.B. (2002). Multiple transcription initiation sites, alternative splicing, and differential polyadenylation contribute to the complexity of human neurofibromatosis 2 transcripts. Genomics.

[19] Gutmann D.H., Sherman L., Seftor L., Haipek C., Hoang Lu K., Hendrix M. (1999). Increased expression of the *NF2* tumor suppressor gene product, merlin, impairs cell motility, adhesionand spreading. Hum. Mol. Genet..

[20] Sherman L., Xu H.M., Geist R.T., Saporito-Irwin S., Howells N., Ponta H., Herrlich P., Gutmann D.H. (1997). Interdomain binding mediates tumor growth suppression by the *NF2* gene product. Oncogene.

[21] Lallemand D., Saint-Amaux A.L., Giovannini M. (2009). Tumor-suppression functions of merlin are independent of its role as an organizer of the actin cytoskeleton in Schwann cells. J. Cell Sci..

[22] Zoch A., Mayerl S., Schulz A., Greither T., Frappart L., Rubsam J., Heuer H., Giovannini M., Morrison H. (2015). Merlin Isoforms 1 and 2 Both Act as Tumour Suppressors and Are Required for Optimal Sperm Maturation. PLoS ONE.

[23] Sher I., Hanemann C.O., Karplus P.A., Bretscher A. (2012). The tumor suppressor merlin controls growth in its open state, and phosphorylation converts it to a less-active more-closed state. Dev. Cell.

[24] Bueno R., Stawiski E.W., Goldstein L.D., Durinck S., De Rienzo A., Modrusan Z., Gnad F., Nguyen T.T., Jaiswal B.S., Chirieac L.R. (2016). Comprehensive genomic analysis of malignant pleural mesothelioma identifies recurrent mutations, gene fusions and splicing alterations. Nat. Genet..

[25] McClatchey A.I., Giovannini M. (2005). Membrane organization and tumorigenesis—The NF2 tumor suppressor, Merlin. Genes Dev..

[26] Morrison H., Sherman L.S., Legg J., Banine F., Isacke C., Haipek C.A., Gutmann D.H., Ponta H., Herrlich P. (2001). The *NF2* tumor suppressor gene product, merlin, mediates contact inhibition of growth through interactions with CD44. Genes Dev..

[27] Sainio M., Zhao F., Heiska L., Turunen O., den Bakker M., Zwarthoff E., Lutchman M., Rouleau G.A., Jaaskelainen J., Vaheri A. (1997). Neurofibromatosis 2 tumor suppressor protein colocalizes with ezrin and CD44 and associates with actin-containing cytoskeleton. J. Cell Sci..

[28] Murthy A., Gonzalez-Agosti C., Cordero E., Pinney D., Candia C., Solomon F., Gusella J., Ramesh V. (1998). NHE-RF, a regulatory cofactor for Na^+^-H^+^ exchange, is a common interactor for merlin and ERM (MERM) proteins. J. Biol. Chem..

[29] Reczek D., Berryman M., Bretscher A. (1997). Identification of EBP50: A PDZ-containing phosphoprotein that associates with members of the ezrin-radixin-moesin family. J. Cell Biol..

[30] Lallemand D., Curto M., Saotome I., Giovannini M., McClatchey A.I. (2003). NF2 deficiency promotes tumorigenesis and metastasis by destabilizing adherens junctions. Genes Dev..

[31] Fehon R.G., McClatchey A.I., Bretscher A. (2010). Organizing the cell cortex: The role of ERM proteins. Nat. Rev. Mol. Cell Biol..

[32] Mani T., Hennigan R.F., Foster L.A., Conrady D.G., Herr A.B., Ip W. (2011). FERM domain phosphoinositide binding targets merlin to the membrane and is essential for its growth-suppressive function. Mol. Cell. Biol..

[33] Hamada K., Shimizu T., Matsui T., Tsukita S., Hakoshima T. (2000). Structural basis of the membrane-targeting and unmasking mechanisms of the radixin FERM domain. EMBO J..

[34] Shimizu T., Seto A., Maita N., Hamada K., Tsukita S., Tsukita S., Hakoshima T. (2002). Structural basis for neurofibromatosis type 2. Crystal structure of the merlin FERM domain. J. Biol. Chem..

[35] Xu H.M., Gutmann D.H. (1998). Merlin differentially associates with the microtubule and actin cytoskeleton. J. Neurosci. Res..

[36] LaJeunesse D.R., McCartney B.M., Fehon R.G. (1998). Structural analysis of *Drosophila* merlin reveals functional domains important for growth control and subcellular localization. J. Cell Biol..

[37] Johnson K.C., Kissil J.L., Fry J.L., Jacks T. (2002). Cellular transformation by a FERM domain mutant of the *NF2* tumor suppressor gene. Oncogene.

[38] Surace E.I., Haipek C.A., Gutmann D.H. (2004). Effect of merlin phosphorylation on neurofibromatosis 2 (*NF2*) gene function. Oncogene.

[39] Rong R., Surace E.I., Haipek C.A., Gutmann D.H., Ye K. (2004). Serine 518 phosphorylation modulates merlin intramolecular association and binding to critical effectors important for NF2 growth suppression. Oncogene.

[40] Jin H., Sperka T., Herrlich P., Morrison H. (2006). Tumorigenic transformation by CPI-17 through inhibition of a merlin phosphatase. Nature.

[41] Shaw R.J., Paez J.G., Curto M., Yaktine A., Pruitt W.M., Saotome I., O’Bryan J.P., Gupta V., Ratner N., Der C.J. (2001). The *NF2* tumor suppressor, merlin, functions in Rac-dependent signaling. Dev. Cell.

[42] Kissil J.L., Wilker E.W., Johnson K.C., Eckman M.S., Yaffe M.B., Jacks T. (2003). Merlin, the product of the *NF2* tumor suppressor gene, is an inhibitor of the p21-activated kinase, Pak1. Mol. Cell.

[43] Xiao G.H., Beeser A., Chernoff J., Testa J.R. (2002). p21-activated kinase links Rac/Cdc42 signaling to merlin. J. Biol. Chem..

[44] Alfthan K., Heiska L., Gronholm M., Renkema G.H., Carpen O. (2004). Cyclic AMP-dependent protein kinase phosphorylates merlin at serine 518 independently of p21-activated kinase and promotes merlin-ezrin heterodimerization. J. Biol. Chem..

[45] Okada T., López-Lago M., Giancotti F.G. (2005). Merlin/*NF-2* mediates contact inhibition of growth by suppressing recruitment of Rac to the plasma membrane. J. Cell Biol..

[46] Laulajainen M., Muranen T., Carpen O., Gronholm M. (2008). Protein kinase A-mediated phosphorylation of the NF2 tumor suppressor protein merlin at serine 10 affects the actin cytoskeleton. Oncogene.

[47] Tang X., Jang S.W., Wang X., Liu Z., Bahr S.M., Sun S.Y., Brat D., Gutmann D.H., Ye K. (2007). AKT phosphorylation regulates the tumour-suppressor merlin through ubiquitination and degradation. Nat. Cell Biol..

[48] Yi C., Troutman S., Fera D., Stemmer-Rachamimov A., Avila J.L., Christian N., Persson N.L., Shimono A., Speicher D.W., Marmorstein R. (2011). A tight junction-associated Merlin-angiomotin complex mediates Merlin’s regulation of mitogenic signaling and tumor suppressive functions. Cancer Cell.

[49] Nguyen R., Reczek D., Bretscher A. (2001). Hierarchy of merlin and ezrin N- and C-terminal domain interactions in homo- and heterotypic associations and their relationship to binding of scaffolding proteins EBP50 and E3KARP. J. Biol. Chem..

[50] Hennigan R.F., Foster L.A., Chaiken M.F., Mani T., Gomes M.M., Herr A.B., Ip W. (2010). Fluorescence resonance energy transfer analysis of merlin conformational changes. Mol. Cell. Biol..

[51] Gutmann D.H., Hirbe A.C., Haipek C.A. (2001). Functional analysis of neurofibromatosis 2 (NF2) missense mutations. Hum. Mol. Genet..

[52] Singhi A.D., Krasinskas A.M., Choudry H.A., Bartlett D.L., Pingpank J.F., Zeh H.J., Luvison A., Fuhrer K., Bahary N., Seethala R.R. (2016). The prognostic significance of BAP1, NF2, and CDKN2A in malignant peritoneal mesothelioma. Mod. Pathol..

[53] Hanahan D., Weinberg R.A. (2011). Hallmarks of cancer: The next generation. Cell.

[54] Bosco E.E., Nakai Y., Hennigan R.F., Ratner N., Zheng Y. (2010). NF2-deficient cells depend on the Rac1-canonical Wnt signaling pathway to promote the loss of contact inhibition of proliferation. Oncogene.

[55] Gladden A.B., Hebert A.M., Schneeberger E.E., McClatchey A.I. (2010). The NF2 tumor suppressor, Merlin, regulates epidermal development through the establishment of a junctional polarity complex. Dev. Cell.

[56] Gonzalez-Agosti C., Xu L., Pinney D., Beauchamp R., Hobbs W., Gusella J., Ramesh V. (1996). The merlin tumor suppressor localizes preferentially in membrane ruffles. Oncogene.

[57] Scherer S.S., Gutmann D.H. (1996). Expression of the neurofibromatosis 2 tumor suppressor gene product, merlin, in Schwann cells. J. Neurosci. Res..

[58] Yokoyama T., Osada H., Murakami H., Tatematsu Y., Taniguchi T., Kondo Y., Yatabe Y., Hasegawa Y., Shimokata K., Horio Y. (2008). YAP1 is involved in mesothelioma development and negatively regulated by Merlin through phosphorylation. Carcinogenesis.

[59] Harvey K.F., Zhang X., Thomas D.M. (2013). The Hippo pathway and human cancer. Nat. Rev. Cancer.

[60] Zanconato F., Cordenonsi M., Piccolo S. (2016). YAP/TAZ at the Roots of Cancer. Cancer Cell.

[61] Tanaka I., Osada H., Fujii M., Fukatsu A., Hida T., Horio Y., Kondo Y., Sato A., Hasegawa Y., Tsujimura T. (2015). LIM-domain protein AJUBA suppresses malignant mesothelioma cell proliferation via Hippo signaling cascade. Oncogene.

[62] Murakami H., Mizuno T., Taniguchi T., Fujii M., Ishiguro F., Fukui T., Akatsuka S., Horio Y., Hida T., Kondo Y. (2011). *LATS2* is a tumor suppressor gene of malignant mesothelioma. Cancer Res..

[63] Kakiuchi T., Takahara T., Kasugai Y., Arita K., Yoshida N., Karube K., Suguro M., Matsuo K., Nakanishi H., Kiyono T. (2016). Modeling mesothelioma utilizing human mesothelial cells reveals involvement of phospholipase-C β4 in YAP-active mesothelioma cell proliferation. Carcinogenesis.

[64] Mizuno T., Murakami H., Fujii M., Ishiguro F., Tanaka I., Kondo Y., Akatsuka S., Toyokuni S., Yokoi K., Osada H. (2012). YAP induces malignant mesothelioma cell proliferation by upregulating transcription of cell cycle-promoting genes. Oncogene.

[65] Li W., You L., Cooper J., Schiavon G., Pepe-Caprio A., Zhou L., Ishii R., Giovannini M., Hanemann C.O., Long S.B. (2010). Merlin/NF2 suppresses tumorigenesis by inhibiting the E3 ubiquitin ligase CRL4(DCAF1) in the nucleus. Cell.

[66] Li W., Cooper J., Zhou L., Yang C., Erdjument-Bromage H., Zagzag D., Snuderl M., Ladanyi M., Hanemann C.O., Zhou P. (2014). Merlin/NF2 loss-driven tumorigenesis linked to CRL4(DCAF1)-mediated inhibition of the Hippo pathway kinases LATS1 and 2 in the nucleus. Cancer Cell.

[67] Cooper J., Xu Q., Zhou L., Pavlovic M., Ojeda V., Moulick K., de Stanchina E., Poirier J.T., Zauderer M., Rudin C.M. (2017). Combined Inhibition of NEDD8-Activating Enzyme and mTOR Suppresses NF2 Loss-Driven Tumorigenesis. Mol. Cancer Ther..

[68] Hikasa H., Sekido Y., Suzuki A. (2016). Merlin/NF2-Lin28B-let-7 Is a Tumor-Suppressive Pathway that Is Cell-Density Dependent and Hippo Independent. Cell Rep..

[69] Saxton R.A., Sabatini D.M. (2017). mTOR Signaling in Growth, Metabolism, and Disease. Cell.

[70] Albert V., Hall M.N. (2015). mTOR signaling in cellular and organismal energetics. Curr. Opin. Cell Biol..

[71] Yang H., Rudge D.G., Koos J.D., Vaidialingam B., Yang H.J., Pavletich N.P. (2013). mTOR kinase structure, mechanism and regulation. Nature.

[72] Sato T., Akasu H., Shimono W., Matsu C., Fujiwara Y., Shibagaki Y., Heard J.J., Tamanoi F., Hattori S. (2015). Rheb protein binds CAD (carbamoyl-phosphate synthetase 2, aspartate transcarbamoylase, and dihydroorotase) protein in a GTP- and effector domain-dependent manner and influences its cellular localization and carbamoyl-phosphate synthetase (CPSase) activity. J. Biol. Chem..

[73] Robitaille A.M., Christen S., Shimobayashi M., Cornu M., Fava L.L., Moes S., Prescianotto-Baschong C., Sauer U., Jenoe P., Hall M.N. (2013). Quantitative phosphoproteomics reveal mTORC1 activates de novo pyrimidine synthesis. Science.

[74] Ben-Sahra I., Howell J.J., Asara J.M., Manning B.D. (2013). Stimulation of de novo pyrimidine synthesis by growth signaling through mTOR and S6K1. Science.

[75] López -Lago M.A., Okada T., Murillo M.M., Socci N., Giancotti F.G. (2009). Loss of the tumor suppressor gene *NF2*, encoding merlin, constitutively activates integrin-dependent mTORC1 signaling. Mol. Cell. Biol..

[76] James M.F., Han S., Polizzano C., Plotkin S.R., Manning B.D., Stemmer-Rachamimov A.O., Gusella J.F., Ramesh V. (2009). NF2/merlin is a novel negative regulator of mTOR complex 1, and activation of mTORC1 is associated with meningioma and schwannoma growth. Mol. Cell. Biol..

[77] Guo Y., Chirieac L.R., Bueno R., Pass H., Wu W., Malinowska I.A., Kwiatkowski D.J. (2014). Tsc1-Tp53 loss induces mesothelioma in mice, and evidence for this mechanism in human mesothelioma. Oncogene.

[78] Altomare D.A., You H., Xiao G.H., Ramos-Nino M.E., Skele K.L., De Rienzo A., Jhanwar S.C., Mossman B.T., Kane A.B., Testa J.R. (2005). Human and mouse mesotheliomas exhibit elevated AKT/PKB activity, which can be targeted pharmacologically to inhibit tumor cell growth. Oncogene.

[79] Suzuki Y., Murakami H., Kawaguchi K., Tanigushi T., Fujii M., Shinjo K., Kondo Y., Osada H., Shimokata K., Horio Y. (2009). Activation of the PI3K-AKT pathway in human malignant mesothelioma cells. Mol. Med. Rep..

[80] Sato T., Nakashima A., Guo L., Coffman K., Tamanoi F. (2010). Single amino-acid changes that confer constitutive activation of mTOR are discovered in human cancer. Oncogene.

[81] Grabiner B.C., Nardi V., Birsoy K., Possemato R., Shen K., Sinha S., Jordan A., Beck A.H., Sabatini D.M. (2014). A diverse array of cancer-associated MTOR mutations are hyperactivating and can predict rapamycin sensitivity. Cancer Discov..

[82] Rehfeld F., Rohde A.M., Nguyen D.T., Wulczyn F.G. (2015). Lin28 and let-7: Ancient milestones on the road from pluripotency to neurogenesis. Cell Tissue Res..

[83] Zhou J., Ng S.B., Chng W.J. (2013). LIN28/LIN28B: An emerging oncogenic driver in cancer stem cells. Int. J. Biochem. Cell Biol..

[84] Johnson S.M., Grosshans H., Shingara J., Byrom M., Jarvis R., Cheng A., Labourier E., Reinert K.L., Brown D., Slack F.J. (2005). RAS is regulated by the let-7 microRNA family. Cell.

[85] Kumar M.S., Lu J., Mercer K.L., Golub T.R., Jacks T. (2007). Impaired microRNA processing enhances cellular transformation and tumorigenesis. Nat. Genet..

[86] Bouwmeester T., Bauch A., Ruffner H., Angrand P.O., Bergamini G., Croughton K., Cruciat C., Eberhard D., Gagneur J., Ghidelli S. (2004). A physical and functional map of the human TNF-α/NF-κB signal transduction pathway. Nat. Cell Biol..

[87] Scudiero I., Zotti T., Ferravante A., Vessichelli M., Reale C., Masone M.C., Leonardi A., Vito P., Stilo R. (2012). Tumor necrosis factor (TNF) receptor-associated factor 7 is required for TNFα-induced Jun NH2-terminal kinase activation and promotes cell death by regulating polyubiquitination and lysosomal degradation of c-FLIP protein. J. Biol. Chem..

[88] Rippo M.R., Moretti S., Vescovi S., Tomasetti M., Orecchia S., Amici G., Catalano A., Procopio A. (2004). FLIP overexpression inhibits death receptor-induced apoptosis in malignant mesothelial cells. Oncogene.

[89] Clark V.E., Erson-Omay E.Z., Serin A., Yin J., Cotney J., Ozduman K., Avsar T., Li J., Murray P.B., Henegariu O. (2013). Genomic analysis of non-NF2 meningiomas reveals mutations in TRAF7, KLF4, AKT1, and SMO. Science.

[90] Shapiro I.M., Kolev V.N., Vidal C.M., Kadariya Y., Ring J.E., Wright Q., Weaver D.T., Menges C., Padval M., McClatchey A.I. (2014). Merlin deficiency predicts FAK inhibitor sensitivity: A synthetic lethal relationship. Sci. Transl. Med..

[91] Liu-Chittenden Y., Huang B., Shim J.S., Chen Q., Lee S.J., Anders R.A., Liu J.O., Pan D. (2012). Genetic and pharmacological disruption of the TEAD-YAP complex suppresses the oncogenic activity of YAP. Genes Dev..

[92] Zhang W.Q., Dai Y.Y., Hsu P.C., Wang H., Cheng L., Yang Y.L., Wang Y.C., Xu Z.D., Liu S., Chan G. (2017). Targeting YAP in malignant pleural mesothelioma. J. Cell. Mol. Med..

[93] Tranchant R., Quetel L., Tallet A., Meiller C., Renier A., de Koning L., de Reynies A., Le Pimpec-Barthes F., Zucman-Rossi J., Jaurand M.C. (2017). Co-occurring Mutations of Tumor Suppressor Genes, *LATS2* and *NF2*, in Malignant Pleural Mesothelioma. Clin. Cancer Res..

[94] Shimada K., Skouta R., Kaplan A., Yang W.S., Hayano M., Dixon S.J., Brown L.M., Valenzuela C.A., Wolpaw A.J., Stockwell B.R. (2016). Global survey of cell death mechanisms reveals metabolic regulation of ferroptosis. Nat. Chem. Biol..

[95] Song S., Xie M., Scott A.W., Jin J., Ma L., Dong X., Skinner H.D., Johnson R.L., Ding S., Ajani J.A. (2018). A Novel YAP1 Inhibitor Targets CSC-Enriched Radiation-Resistant Cells and Exerts Strong Antitumor Activity in Esophageal Adenocarcinoma. Mol. Cancer Ther..

[96] Lin K.C., Moroishi T., Meng Z., Jeong H.S., Plouffe S.W., Sekido Y., Han J., Park H.W., Guan K.L. (2017). Regulation of Hippo pathway transcription factor TEAD by p38 MAPK-induced cytoplasmic translocation. Nat. Cell Biol..

[97] Ou S.H., Moon J., Garland L.L., Mack P.C., Testa J.R., Tsao A.S., Wozniak A.J., Gandara D.R. (2015). SWOG S0722: Phase II study of mTOR inhibitor everolimus (RAD001) in advanced malignant pleural mesothelioma (MPM). J. Thorac. Oncol..

[98] Thoreen C.C., Kang S.A., Chang J.W., Liu Q., Zhang J., Gao Y., Reichling L.J., Sim T., Sabatini D.M., Gray N.S. (2009). An ATP-competitive mammalian target of rapamycin inhibitor reveals rapamycin-resistant functions of mTORC1. J. Biol. Chem..

[99] Agarwal V., Campbell A., Beaumont K.L., Cawkwell L., Lind M.J. (2013). PTEN protein expression in malignant pleural mesothelioma. Tumour Biol..

[100] Goel S., Wang Q., Watt A.C., Tolaney S.M., Dillon D.A., Li W., Ramm S., Palmer A.C., Yuzugullu H., Varadan V. (2016). Overcoming Therapeutic Resistance in HER2-Positive Breast Cancers with CDK4/6 Inhibitors. Cancer Cell.

[101] Franco J., Balaji U., Freinkman E., Witkiewicz A.K., Knudsen E.S. (2016). Metabolic Reprogramming of Pancreatic Cancer Mediated by CDK4/6 Inhibition Elicits Unique Vulnerabilities. Cell Rep..

[102] Bonelli M.A., Digiacomo G., Fumarola C., Alfieri R., Quaini F., Falco A., Madeddu D., La Monica S., Cretella D., Ravelli A. (2017). Combined Inhibition of CDK4/6 and PI3K/AKT/mTOR Pathways Induces a Synergistic Anti-Tumor Effect in Malignant Pleural Mesothelioma Cells. Neoplasia.

[103] Rodrik-Outmezguine V.S., Okaniwa M., Yao Z., Novotny C.J., McWhirter C., Banaji A., Won H., Wong W., Berger M., de Stanchina E. (2016). Overcoming mTOR resistance mutations with a new-generation mTOR inhibitor. Nature.

[104] Fan Q., Aksoy O., Wong R.A., Ilkhanizadeh S., Novotny C.J., Gustafson W.C., Truong A.Y., Cayanan G., Simonds E.F., Haas-Kogan D. (2017). A Kinase Inhibitor Targeted to mTORC1 Drives Regression in Glioblastoma. Cancer Cell.

[105] Demierre M.F., Higgins P.D., Gruber S.B., Hawk E., Lippman S.M. (2005). Statins and cancer prevention. Nat. Rev. Cancer.

[106] Rubins J.B., Greatens T., Kratzke R.A., Tan A.T., Polunovsky V.A., Bitterman P. (1998). Lovastatin induces apoptosis in malignant mesothelioma cells. Am. J. Respir. Crit. Care Med..

[107] Asakura K., Izumi Y., Yamamoto M., Yamauchi Y., Kawai K., Serizawa A., Mizushima T., Ohmura M., Kawamura M., Wakui M. (2011). The cytostatic effects of lovastatin on ACC-MESO-1 cells. J. Surg. Res..

[108] Yamauchi Y., Izumi Y., Asakura K., Fukutomi T., Serizawa A., Kawai K., Wakui M., Suematsu M., Nomori H. (2011). Lovastatin and valproic acid additively attenuate cell invasion in ACC-MESO-1 cells. Biochem. Biophys. Res. Commun..

[109] Tuerdi G., Ichinomiya S., Sato H., Siddig S., Suwa E., Iwata H., Yano T., Ueno K. (2013). Synergistic effect of combined treatment with gamma-tocotrienol and statin on human malignant mesothelioma cells. Cancer Lett..

[110] Hwang K.E., Kim Y.S., Hwang Y.R., Kwon S.J., Park D.S., Cha B.K., Kim B.R., Yoon K.H., Jeong E.T., Kim H.R. (2014). Enhanced apoptosis by pemetrexed and simvastatin in malignant mesothelioma and lung cancer cells by reactive oxygen species-dependent mitochondrial dysfunction and Bim induction. Int. J. Oncol..

[111] Tanaka K., Osada H., Murakami-Tonami Y., Horio Y., Hida T., Sekido Y. (2017). Statin suppresses Hippo pathway-inactivated malignant mesothelioma cells and blocks the YAP/CD44 growth stimulatory axis. Cancer Lett..

[112] Wang Z., Wu Y., Wang H., Zhang Y., Mei L., Fang X., Zhang X., Zhang F., Chen H., Liu Y. (2014). Interplay of mevalonate and Hippo pathways regulates RHAMM transcription via YAP to modulate breast cancer cell motility. Proc. Natl. Acad. Sci. USA.

[113] Sorrentino G., Ruggeri N., Specchia V., Cordenonsi M., Mano M., Dupont S., Manfrin A., Ingallina E., Sommaggio R., Piazza S. (2014). Metabolic control of YAP and TAZ by the mevalonate pathway. Nat. Cell Biol..

[114] Guerrant W., Kota S., Troutman S., Mandati V., Fallahi M., Stemmer-Rachamimov A., Kissil J.L. (2016). YAP Mediates Tumorigenesis in Neurofibromatosis Type 2 by Promoting Cell Survival and Proliferation through a COX-2-EGFR Signaling Axis. Cancer Res..

[115] Wahle B.M., Hawley E.T., He Y., Smith A.E., Yuan J., Masters A.R., Jones D.R., Gehlhausen J.R., Park S.J., Conway S.J. (2018). Chemopreventative celecoxib fails to prevent schwannoma formation or sensorineural hearing loss in genetically engineered murine model of neurofibromatosis type 2. Oncotarget.

[116] Yap T.A., Aerts J.G., Popat S., Fennell D.A. (2017). Novel insights into mesothelioma biology and implications for therapy. Nat. Rev. Cancer.

[117] Guo X.E., Ngo B., Modrek A.S., Lee W.H. (2014). Targeting tumor suppressor networks for cancer therapeutics. Curr. Drug Targets.

[118] Cooper J., Giancotti F.G. (2014). Molecular insights into NF2/Merlin tumor suppressor function. FEBS Lett..

[119] McCambridge A.J., Napolitano A., Mansfield A.S., Fennell D.A., Sekido Y., Nowak A.K., Reungwetwattana T., Mao W., Pass H.I., Carbone M. (2018). State of the art: Advances in Malignant Pleural Mesothelioma in 2017. J. Thorac. Oncol..

[120] Shalem O., Sanjana N.E., Hartenian E., Shi X., Scott D.A., Mikkelson T., Heckl D., Ebert B.L., Root D.E., Doench J.G. (2014). Genome-scale CRISPR-Cas9 knockout screening in human cells. Science.

